# Factors Associated with Health-Related Quality of Life in Women with Breast Cancer in the Middle East: A Systematic Review

**DOI:** 10.3390/cancers12030696

**Published:** 2020-03-16

**Authors:** Rana El Haidari, Linda Abou Abbas, Virginie Nerich, Amélie Anota

**Affiliations:** 1 Environments and Health doctoral school, University of Bourgogne Franche-Comté, 25000 Besançon, France; 2 INSERM (French Institut of Health and Medical Research), EFS BFC (Etablissement français du sang Bourgogne franhce-comté), UMR1098 (Interactions Greffon-Hôte-Tumeur/Ingénierie Cellulaire et Génique), University of Bourgogne Franche-Comté, RIGHT Interactions Greffon-Hôte-Tumeur/Ingénierie Cellulaire et Génique, F-25000 Besançon, France; v1nerich@chu-besancon.fr (V.N.); aanota@chu-besancon.fr (A.A.); 3 Neuroscience research center, Faculty of medical sciences, Lebanese university, 1001 Beirut, Lebanon; l.abouabbas@ul.edu.lb; 4 Department of Pharmacy, University Hospital of Besançon, 25030 Besançon, France; 5 Methodology and Quality of Life in Oncology Unit, University Hospital of Besançon, 25000 Besançon, France; 6 French National Platform Quality of Life and Cancer, 250000 Besançon, France

**Keywords:** Breast Cancer, Health-related quality of life, Systematic review, Middle East, Women

## Abstract

Objectives: The aim of the present systematic review was to identify the factors that potentially influence health-related quality of life (HRQoL) in women with breast cancer (BC) in the Middle East. Methods: A systematic search of the PubMed, Ovid Medline, Cochrane, Embase, Cumulative Index to Nursing and Allied Health Literature (CINAHL), Scopus, and Ebscohost databases was conducted to identify all relevant articles published in peer-reviewed journals up to April 2018. The keywords were “Health related quality of life”, “Breast Cancer”, and “Middle East countries”. The Newcastle–Ottawa (NOS) scale was used to evaluate the methodological quality of the included studies. Due to the methodological heterogeneity of the identified studies, no statistical pooling of the individual effect estimates was carried out; instead, the results were summarized descriptively. Results: A total of 5668 articles were screened and 33 studies were retained. The vast majority of these studies were cross-sectional and only two were longitudinal prospective studies. Concerning the methodological quality, only 39% were of high quality. Our comprehensive literature review identified several modifiable and non-modifiable risk factors associated with HRQoL, including sociodemographic, clinical, and treatment-related factors as well as behavioral and psychosocial factors. Conclusion: This study has many implications for clinical practice and may provide a framework for establishing policy interventions to improve HRQoL among women with BC. Healthcare systems in the Middle East are encouraged to develop interventional programs targeting modifiable factors, particularly socio-demographic, behavioral, and psychosocial factors.

## 1. Introduction

Globally, breast cancer (BC) is the most commonly occurring cancer in women [1]. The worldwide GLOBOCAN estimates revealed that 2,088,849 new BC cases and 626,679 cancer-related deaths occurred in 2018, with a projected number of over 3,059,829 women to be diagnosed in 2040 [1]. This reflects a significant global increase of 46%, albeit with significant differences in incidence between countries [1].

The Middle East is a region located in western Asia and extends into North Africa, with an estimated population of over 411 million. It comprises 15 countries with different ethnic groups holding different cultures, norms, and beliefs. Although BC incidence rates are lower in the Middle East compared to other western societies, they have substantially increased over the last few decades [1]. According to GLOBOCAN estimates, approximately 119,985 (34.2%) new BC cases and 48,661 (24.9%) cancer-related deaths occurred in 2018 [1]. Furthermore, a large proportion of women are diagnosed with BC at younger age, i.e., under 40 years old, and at an advanced stage, where BC is generally aggressive and requires mastectomy [2]. In most countries of the Middle East, BC is still considered a sensitive and taboo topic, surrounded by shame and silence. Most Middle Eastern societies mention it as ‘‘that other disease’’ and many women are petrified to talk about it [3]. The perception of BC as incurable and its fatality are also a concern. Therefore, a diagnosis of BC can be a devastating event and can expose Middle Eastern women to a high burden of psychological suffering that could adversely affect their health-related quality of life (HRQoL).

According to Osoba et al., HRQoL is “a multidimensional construct that includes perceptions of both positive and negative aspects of dimensions such as physical, emotional, social and cognitive functions. It also includes the negative aspects of somatic discomfort and other symptoms produced by a disease or its treatment” [4]. HRQoL is considered one of the main determinants of treatment success in modern oncology [5,6]. Findings from research suggest that assessment of HRQoL as part of clinical practice has the potential to improve the quality of care that patients receive, as well as their health status. Thus, its evaluation has become as important as survival in making treatment decisions [7].

Over the last few decades, evaluation of HRQoL and its associated factors in patients diagnosed with BC have witnessed increasing interest among both researchers and decision-makers [8]. Studies from western societies have identified several factors affecting HRQoL in women with BC. Those factors include socio-demographic characteristics, such as age [9], marital status, economic problems [10], and the experience of daily difficulties in work [11], as well as clinical and psychological characteristics of BC survivors, such as stage of disease [12], pain, stress [13], depression and anxiety [14], social relationships [15], variations in self-confidence, self-efficacy [16], and the development of harmful sentiments. In addition, HRQoL was found to be affected by the therapeutic procedures used [17].

A recent review and meta-analysis evaluating the QoL of patients with BC in the Middle East revealed that fewer than one-third of patients (21%) had good QoL [18]. However, studies on factors associated with HRQoL in patients with BC in Middle Eastern countries are scarce. Findings from western societies cannot be extrapolated to BC patients in the Middle East countries, which have a distinct lifestyle and culture. Several potential issues may contribute to the expected disparity, including socioeconomic difficulties, limited access to health care, lack of supportive care, late BC diagnosis, self-perception of the disease, social constraints, and other religious/cultural restrictions [3]. Identification of the factors associated with HRQoL could help professionals to develop effective health interventions and specific approaches to promote quality of life in BC patients in the Middle East. Therefore, there is a need for a synthesis of existing research to aid in the design of interventions to improve HRQoL. In this context, the aim of the present study is to perform a systematic review to identify the factors associated with HRQoL in the countries of the Middle East.

## 2. Materials and Methods

The systematic review was performed in accordance with the Preferred Reporting Items for Systematic reviews and Meta-Analyses guidelines (PRISMA) published in 2009 [19]. An ethics statement was not required.

### 2.1. Data Sources and Search Strategy

A systematic search of the following databases was conducted: PubMed, Ovid Medline, Cochrane, Embase, Cumulative Index to Nursing and Allied Health Literature (CINAHL), and Ebscohost, to identify all relevant articles published in peer-reviewed journals up to April 2018. Keywords related to “Health related quality of life”, “Breast Cancer”, and “Middle East countries” were combined using Boolean operators (“AND”, and “OR”). The search combination of keywords and Medical Subject Headings (MeSH) terms is presented in Table 1. Bibliography lists from all eligible articles were also hand searched to identify additional papers potentially relevant for inclusion.

### 2.2. Eligibility Criteria

Articles were included if they met all of the following criteria:

1. Population: women diagnosed with BC only;
2. Setting: studies conducted in one or more Middle East countries;
3. Outcome: HRQoL assessed using any valid and reliable instrument that measures quality of life, such as EORTC QLQ-C30: European Organization for Research and treatment of Cancer—Quality of Life Questionnaire; BR23: Breast cancer specific module; FACT-B: The Functional Assessment of Cancer Therapy—Breast Cancer; FACT-G: The Functional Assessment of Cancer Therapy—General Questionnaire; NMCBRI-Q: the national medical center and Beckman research institute questionnaire; LQI: Life quality inventory; QOL-BC: The American quality of life BC; QoLS: The Quality of Life Scale; SF-36: Short-Form Health Survey Questionnaire; WHOQOL-BREF: WHO questionnaire of quality of life;
4. Experimental or observational study investigating any associated factors with HRQoL;
5. English language articles.

Articles were excluded if they met any one or more of the following criteria:

1. Narrative or systematic reviews;
2. Editorials, expert opinions, comments (commentary), methodological article, or conference abstracts and proceedings;
3. Assessment of the effect of specific interventions on HRQoL, such as sport, exercise, yoga, or focus groups.

### 2.3. Data Extraction and Synthesis

Titles and abstracts identified from searches were screened for relevance, and duplicates were excluded. The full texts of all relevant articles were retrieved and their eligibility for inclusion was assessed.

Two reviewers (R.H. and L.A.A.) performed data extraction independently. Any disagreements were resolved by discussion and by cross-checking the papers. The following information was recorded from each paper: basic study information (last author’s name, publication year, and country), study design, sample characteristics (sample size and age), clinical data (BC stage and treatment), HRQoL questionnaire used and time of assessment, as well as the results of the studies, including global HRQoL (Mean and SD), determinants, and signs of the association.

### 2.4. Methodological Quality Assessment

The methodological quality and risk of bias of the included studies was assessed independently by two reviewers (R.H. and L.A.A.) using the Newcastle–Ottawa scale (NOS), a quality assessment tool [20]. Ratings are made using a “star” system to assess the quality of a study for eight items grouped into three domains: (1) selection of participants, (2) comparability of study groups, and (3) verification of the exposure or the outcome of interest. According to the NOS scale, a maximum of nine stars can be allocated for case-control or longitudinal studies and a maximum of six stars can be obtained for cross-sectional studies. Case-control or longitudinal studies that score five or more stars and cross-sectional studies with four or more stars are considered to be of high quality.

## 3. Results

### 3.1. Study Selection

**Figure 1:**
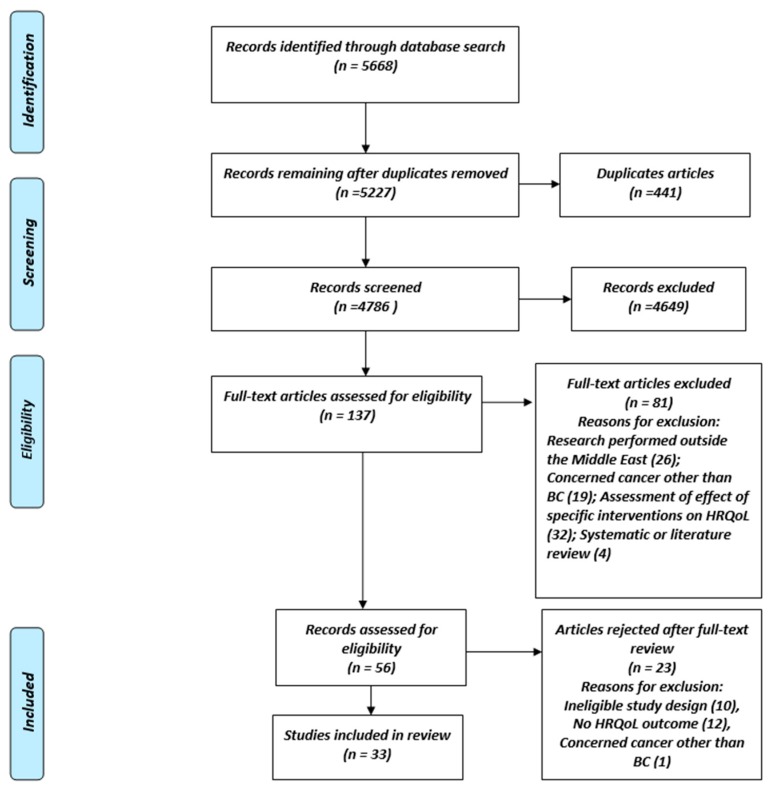
Flow chart of study selection strategy.

The literature search identified a total of 5668 records. After the exclusion of duplicate records and articles with non-relevant title and abstracts, 5227 published studies were retained for screening. Screening of titles and abstracts identified 137 potentially eligible articles. Full-text analyses of these 137 articles identified 56 relevant articles, of which 33 finally met the eligibility criteria for inclusion in the present review (Figure 1).

### 3.2. Characteristics of the Included Studies

The characteristics of the articles included in the present systematic review are summarized in Table 2. Almost half of the studies were conducted in Iran (*n* = 15, 45%), followed by Turkey (*n* = 9, 27%), Saudi Arabia (*n* = 3, 9%), Jordan (*n* = 2, 6%), and one each (3%) from Lebanon, Kuwait, Bahrain, and Yemen (Table 2). More than half of the studies in were from Cancer Centers (*n* = 17, 52%) and the rest (*n* = 16, 48%) were conducted in hospitals. First authors of the published studies were mainly affiliated with universities. Articles were published in medical (42%) or medical oncology journals (18%). About two-thirds of the studies (64%) used interviews as a method to collect data. Concerning study designs, the majority had a cross-sectional design (*n* = 28, 85%). Three studies used a case-control design, while only two were longitudinal studies.

### 3.3. Description of the Selected Atudies

A detailed description of the included studies is presented in Table 3. The majority of the women participating in the studies had stage II BC and were treated with chemotherapy.

#### 3.3.1. Characteristics of the Cross-Sectional Studies

Sample sizes of the included cross-sectional studies (*n* = 28) ranged from 42 to 762 patients, and totaled 8764 women with BC included in all studies. Age ranged from 25 to 60 years. The studies included were published between 2004 and 2018. For outcome assessment, specific BC questionnaires, such as the European Organization for Research and treatment of Cancer—Quality of Life Questionnaire (EORTC QLQ-C30) associated with the breast cancer module (QLQ-BR23) (*n* = 8), the Functional Assessment of Cancer Therapy-Breast Cancer (FACT-B) (*n* = 4), as well as generic tools were used.

#### 3.3.2. Characteristics of the Case-Control Studies

The total number of women with BC in case-control studies was 318, with a mean age ranging from 32 to 49 years. These studies were published between 2015 and 2017, and were reported from Iran. One study used the EORTC QLQ-C30, the second one used the EORTC QLQ-C30 associated with the QLQ-BR23, and the third used the generic SF-36 questionnaire.

#### 3.3.3. Characteristics of the Longitudinal Studies

Concerning the two longitudinal studies, a total number of 241 BC women with mean ages of 46.9 and 47.2 years were included. The two studies were published in 2008 and were reported from Turkey and Iran. With regard to the instruments used to assess HRQoL, one study used the FACT-B and the other used the EORTC QLQ-C30.

### 3.4. Quality Assessment

A summary of the quality assessment results is presented in Table 4a,b. According to quality criteria on the NOS scale, ten cross-sectional studies were classed as high quality (five stars or more out of six). Two case-control studies and one longitudinal study were of high quality (six stars out of nine).

### 3.5. Factors Associated with HRQoL in Women with BC in the Middle East

The authors of the included studies identified several factors associated with global HRQOL in women with BC. Table 5 presents a summary of these factors.

#### 3.5.1. Socio-Demographic Factors

Several socio-demographic factors were closely associated with HRQoL in patients with BC. These included: age [21,24,41,43,45,49], marital status [24,37], level of education [22,31,40,44,51], employment status [47,48,51], income [22,23,24,25,28,39,48], and having children [37]. Regarding age, the direction of the association was not consistent across studies, with three studies reporting a negative association with HRQoL [21,24,49], while three other studies reported the opposite [41,43,45]. Being employed, having a higher level of education, and having children were factors found to be associated with better HRQoL. Conversely, being married, having a low income, and financial difficulties adversely affected HRQoL.

#### 3.5.2. Clinical Factors

Multiple clinical factors, including multiple tumors [21], cancer stage [23,24,27,31,32,35,48], metastasis [21], time since diagnosis [24,28,32,45], time since operation [32], disease duration [45], menopausal status [35,48], higher symptom score on the Memorial Symptom Assessment Scale [33], Eastern cooperative oncology group performance score [30], fever [21], pain [25,34,36], fatigue [40,49], and dyspnea [48,49] were found to be associated with HRQoL.

#### 3.5.3. Treatment-Related Factors

HRQoL was negatively influenced by chemotherapy, including Docetaxel with doxorubicin and cyclophosphamide/Gemcitabine + cisplatin/FAC/FEC and Docetaxel/Paclitaxel AC/EC in four studies [23,24,31,37] and by breast-sparing surgery in one study [24], whereas other studies reported that HRQoL was positively influenced by hormone therapy [49], early treatment [45], and breast reconstruction surgery [50]. One study found a positive association with radiotherapy [28], while another reported the reverse [49]. HRQoL was positively affected by the use of complementary alternative medicine [26].

#### 3.5.4. Behavioral Factors

Behavioral factors such as physical activity [21], fitness orientation and evaluation [30], body weight [24,29], and nutritional status [38] were reportedly associated with HRQoL, with women of normal weight and exercising regularly, and well-nourished women having better HRQoL. In addition, positive body image [30], body satisfaction [30], as well as positive religiosity [52,53] and spiritual well-being [29,34] were associated with better HRQoL.

#### 3.5.5. Psychosocial Factors

Several psychosocial factors were reported to have a significant impact on HRQoL. Depression [23,46], anxiety [23], psychological symptoms (including feeling nervous, feeling sad, worrying, difficulty sleeping, “I don’t look like myself”) [33], emotional functioning, helplessness coping [32], and unmet needs [47] had significant negative impacts on HRQoL. Conversely, self-efficacy [39], self-regulation [42], sense of coherence [44], and emotion focus coping strategy [36] had positive impacts on HRQoL.

## 4. Discussion

The main aim of the present review was to synthesize the literature exploring the factors that influence HRQoL in women with breast cancer (BC) in the Middle East. After an extensive literature review, we identified only 33 articles that met our inclusion criteria. The vast majority of these studies were cross-sectional and only three studies were longitudinal prospective studies. In almost 60%, studies were published in medical journals, readily available for both medical practitioners and decision-makers. The methodological quality was high in only 39% of the studies, indicating that there was a high risk of biased results. Our comprehensive literature review identified several sociodemographic, clinical, and treatment-related factors, as well as behavioral and psychosocial factors associated with HRQoL. These findings provide a scientific basis to develop a comprehensive multidimensional program that incorporates these factors, to improve the QoL of breast cancer survivors in the Middle East.

Concerning sociodemographic factors, there was an inconsistency between studies regarding age. The results of three studies suggested that HRQoL was adversely affected in older patients, while three other studies found that younger patients with BC experienced poorer HRQoL than their older counterparts. This discrepancy may be explained by the heterogeneity of the samples, the subjects included, and a lack of power due to the low sample size. Thus, we cannot draw any clear conclusion about the effect of age on HRQoL and further studies are needed to evaluate the association between these two variables. The results of the studies reviewed here consistently suggested that highly educated woman had better quality of life compared to their less well-educated counterparts. A possible explanation for this finding is the ability of educated women to understand the nature of the disease and to comply with the therapeutic recommendations better than the less educated. Moreover, illiterate women with low income are less likely to be screened for breast cancer, delay before seeking care in the presence of symptoms, and are diagnosed at later stages of the disease. Therefore, health care teams should give more attention and support to less well-educated women (i.e., less than secondary level and illiterate individuals) with BC who need extensive information about their treatment and follow-up.

Regarding socioeconomic status, the studies in this review consistently found that patients who were unemployed and had financial difficulties or a lower monthly income reported lower HRQoL than patients who had higher incomes or no financial difficulties [54,55]. In fact, higher economic status can be linked to many aspects of improved patient care, such as rapid access to treatment and rehabilitation, as well as less concern for the financial burden of the treatment [54]. Being married was also found to be associated with better HRQoL in BC patients. In line with this finding, studies from the US and China have found that married BC patients had better QoL compared to single or divorced women [54,56]. This could be explained by the emotional support provided by their spouses. Finally, having children was also found to be associated with better HRQoL, although the number of studies was insufficient to draw clear conclusions.

Regarding clinical and treatment-related factors, HRQoL was significantly impaired by the type of treatment, by advanced stages of disease, and by the symptoms experienced. Chemotherapy was consistently associated with poorer HRQoL in Middle Eastern women with BC. Indeed, patients on chemotherapy are more likely to be diagnosed with advanced stage disease and to experience pain, fatigue, and possibly other severe side effects, which in turn reduce quality of life. Other treatments, such as hormone therapy and breast reconstruction surgery, are less likely to be associated with advance stage disease, and thus less likely to negatively affect quality of life. Regarding radiotherapy, findings were conflicting across studies, and no clear conclusion emerged. Interestingly, one of the studies reviewed [26] reported that complementary and alternative medicine, including spiritual therapy, honey, olive oil, and herbal therapy, was associated with better global HRQoL, physical role, and social functioning, as well as alleviating cancer-related constipation. Since this type of medicine is commonly used in the Middle East, these findings warrant confirmation with a view to incorporating them into medical care and management programs for breast cancer patients.

Concerning behavioral factors, well-nourished women [38] with normal weight [24,29] and those who exercise regularly [21] tended to have better quality of life. In line with these findings, Gong et al. reported a positive effect of physical exercise and healthy diet on HRQoL [54,56]. Thus, promoting patient participation in rehabilitation programs, including nutritional education and physical exercise, might be one way to improve HRQoL in patients with BC. Moreover, consistent with previous findings reported by Wildes et al. [57] our literature review revealed that positive religiosity [52,53] and spiritual well-being were associated with better HRQoL. However, body image disturbance and dissatisfaction were found to be associated with poor HRQoL. In fact, body image disturbance following treatment of cancer may be associated with a variety of changes, such as depression and anxiety, that can have a significant negative impact on HRQoL. Therefore, it is important to evaluate body weight perception in BC patients after chemotherapy or mastectomy, as this may affect biopsychosocial functioning [42].

Several psychosocial factors were found to be associated with HRQoL. As expected, depression and anxiety had a significant negative impact on HRQoL. This is in agreement with the findings of Poleshuck et al. and Shelby et al., who found that patients with BC may experience anxiety and depression regarding surgical experience, coping with acute pain, treatment regimens, financial burden of care, and disruptions to their personal and professional lives [58,59]. All of these factors may in turn adversely affect their quality of life. Thus, early identification and interventions to alleviate depression, anxiety, and stress may help improve HRQoL. It was also found throughout the literature review that having higher scores of self-efficacy [39], self-regulation [42], and sense of coherence had a positive impact on HRQoL. As psychosocial factors are considered to be modifiable, there may be substantial gains to be yielded from paying greater attention to these factors, with a view to improving HRQoL among patients with BC.

This systematic review of 33 studies totaling 5735 participants is the first systematic review to investigate the factors associated with HRQOL in women with BC in the Middle East. Our review has, however, several limitations which need to be considered with caution when interpreting the results. First, despite the rigorous and extensive search strategy with no restrictions on year of publication, there may have been some potentially relevant studies that were eligible, but excluded, because we limited our review to studies published in English. As in any systematic review, publication bias may have affected our findings. Second, the lack of data for some countries may decrease the generalizability of findings to all Middle East regions. Third, due to the heterogeneity among the studies included in the review, only a narrative review was possible. Fourth, the majority of studies had a cross-sectional design, and small sample sizes, implying a low level of evidence, and as such cannot be used to determine causal mechanisms. Finally, although based on the best available data, our review was limited by the quality of studies reported from the Middle Eastern countries.

Despite these limitations, the current review addressed critical factors that were significantly associated with HRQoL in patients with BC. Emphasis should be given to empowering women through education, as this is a key tool for avoiding unemployment and tackling the psychological impact of BC. Financial aids may also significantly improve the HRQoL of BC patients. Thus, healthcare systems in the Middle East are encouraged to expand interdisciplinary palliative and supportive care services that have the necessary expertise to help financially strained patients to navigate the BC care pathway. Moreover, there is also a compelling need to provide social support over the long-term to patients with BC. We recommend that clinicians pay attention to modifiable risk factors that have an influence on HRQoL, such as psychological factors. They should also encourage their patients to strengthen their social relationships with family members and friends, to adhere to a healthy diet, and to practice any kind of sport.

## 5. Conclusions

In summary, the present study identified several modifiable and non-modifiable factors that affect HRQoL in women with BC in the Middle East. This study has many implications for practice and provides a framework for establishing policy interventions to more efficiently improve the QoL of women with BC. Healthcare systems in the Middle East are encouraged to develop targeted interventional programs on modifiable factors, particularly socio-demographic, behavioral, and psychosocial ones. Further research on these factors is warranted, preferably through prospective longitudinal studies.

## Figures and Tables

**Table 1:** Medical Subject Headings (MeSH) terms used in the search strategy.

MeSH Terms for HRQoL	MeSH Terms for Breast Cancer	MeSH Terms for ME Countries
quality of life OR health related quality of life OR life style OR well-being OR health status	Breast Neoplasms OR Breast Neoplasm OR Breast Cancer OR Breast Carcinoma OR Breast OR mammary carcinoma OR cancer OR mammary cancer OR carcinoma mammae	Middle East OR Bahrain OR Egypt OR Iran OR Iraq OR Jordan OR Kuwait OR Lebanon OR Oman OR Palestine OR Qatar OR Saudi Arabia OR Syria OR Turkey OR United Arab Emirates OR Yemen

**Table 2:** Characteristics of the 33 studies included in the systematic review.

Characteristic	Number	Percentage
Country		
Iran	15	45
Turkey	9	27
Saudi Arabia	3	9
Jordan	2	6
Lebanon	1	3
Kuwait	1	3
Bahrain	1	3
Yemen	1	3
Location of study		
Cancer center	17	52
Hospital	16	48
Main location of first author		
University	27	82
Hospital	3	9
Research center	2	6
Medical center	1	3
Type of journal		
Medical	14	42
Medical oncology	6	18
Multidisciplinary	4	12
Clinical	4	12
Academic	4	12
Clinical psychology	1	3
Type of data collection		
Self-reported	12	36
Interview	21	64
Study design		
Cross-sectional	28	85
Case-control	3	9
Longitudinal	2	6

**Table 3:** Description of the selected studies.

Author Name, Country—Year of Publication	Type of Study	Sample Size	Age of BC Patients	Stage	Treatment	Time of Assessment	Questionnaires
			Mean (SD)	%	%		
Ahmad A [21], Saudi Arabia—2017	Cross-sectional	145	50.3 (13.5)	I and II: 61.2 III and IV: 38.8	Cancer surgery: 64.8 Chemotherapy: 73.1 Radiotherapy: 57.9 Immunotherapy: 49	First year after cancer % Yes: 52.4 No: 47.6	SF-36
Ahrafizadeh H [22], Iran—2017	Cross-sectional	100	46.8 (11.5)	-	Mastectomy: 58 Breast conservative surgery: 31 Chemotherapy: 100	During chemotherapy	WHOQOL-BREF
Akel R [23], Lebanon—2017	Cross-sectional	150 (121 Lebanese, 26 Iraqi, 6 other)	53.5 (10.4)	I: 29.3 II: 38.7 III: 23.3 IV: 8.7	Chemotherapy: 79.3 Radiotherapy: 80.7 Surgery: 97.3 Hormone therapy: 86	0.5 to ≤ 2 years: 13.3 3 to ≤ 5 years: 68.7 ≥ 5 years: 18	FACT-B
Akin S [24], Turkey—2008	Longitudinal	141	46.9 (10.1)	I: 6.4 II: 47.5 III: 31.2 IV: 14.29	Chemotherapy: 100 Surgery: 76.6	Pre and Post chemotherapy	FACT-B
Alawadi S [25], Kuwait—2009	Cross-sectional	348	48.3 (10.3)	I: 7 II: 34.3 III: 36 IV: 22.7	Chemotherapy: 98.3 Radiotherapy: 21 Surgery: 58.6	During chemotherapy	EORTC-QLQ-C30 plus QLQ-BR23
Albabtain H [26], Saudi Arabia—2018	Cross-sectional	95	25–39 years: 26.3 40–59 years: 47.4 > 60 years: 26.3	-	89.5 undergoing cancer treatment	During cancer treatment	EORTC-QLQ-C30 plus QLQ-BR23
Almutairi K [27], Saudi Arabia—2016	Cross-sectional	145	26–35 years: 13.1 36–45 years: 30.3	I: 21.4 II: 57.9 III and IV: 20 Distant metastasis: 0.7	Chemotherapy: 3.4 Radiotherapy: 2.1 Surgery: 31.7 Hormone therapy: 0.7 Combination modalities: 62.1	During the visit of outpatient clinics	EORTC-QLQ-C30 plus QLQ-BR23
			>46 years: 56.6				
Al-Naggar R [28], Yemen—2011	Cross-sectional	106	< 55 years: 67.9	III: 35.8	Chemotherapy: 94.3 Radiotherapy: 63.2 Surgery: 85.8	unspecified	FACT-B
Al-Natour A [29], Jordan—2017	Cross-sectional	150	47.9 (9.7)	I: 25.5 II: 48.9 III: 19 IV: 6.6	-	During cancer treatment	FACT-G
Bagheri M [30], Iran—2015	Case- Control	50 cases 50 Healthy control	Cases: 32 (0.5) Controls: 33.7 (4.2)	-	-	unspecified	SF-36
Bayram Z [31], Turkey—2014	Cross-sectional	105	50.1 (11.8)	I: 40 II: 37.1 III: 19.0 IV: 3.8	Chemotherapy: 100	Newly diagnosed and undergoing chemotherapy	FACT-G
Filazoglu G [32], Turkey—2008	Cross-sectional	188	45.1 (5.6)	I: 23.4 II: 55.9 III: 18.6 IV: 2.1	Chemotherapy: 17.6 Radiotherapy: 38.8 Chemotherapy + Radiotherapy: 14.4 No treatment: 29.3 Surgery: 100	After breast surgery for a minimum of 3 months before the study.	SF-36
Hujeir H [33], Lebanon—2012	Cross-sectional	89	49.2 (11.1)	Metastasis: 37.1	Chemotherapy: 47.2 Radiotherapy: 31.4 Surgery: 70.7 Hormone therapy: 15.7	30.6 (39.06) months	EORTC-QLQ-C30
Jafari N [34], Iran—2013	Cross-sectional	68	48 (10.3)	-	Radiotherapy: 100	During Radiotherapy	EORTC-QLQ-C30 plus QLQ-BR23
Jassim G [35], Bahrain—2013	Cross-sectional	239	50.2 (11.1)	I: 29.9 II: 44.8 III and IV: 25.3	Chemotherapy: 80.5 Radiotherapy: 83.9 Lumpectomy: 51.3 Mastectomy: 50 Lymph node dissection: 85.1 Hormone therapy: 69.8	Early diagnosed: 14.6 Transitional period: 53.6 Long-term survivors: 31.8	EORTC-QLQ-C30 plus QLQ-BR23
Khalili N [36], Iran—2013	Cross-sectional	62	45.8 (6.7)	IA: 1.6 IB: 6.4 IIA: 22.5 IIB: 25.8 IIIA: 16.1 IIIB: 14.5 IIIC: 8.1	-	Diagnosis with BC in a recent year	EORTC-QLQ-C30
Kiadaliri A [37], Iran—2012	Longitudinal	100	TAC: 46.7 (8.2) FAC: 49.3 (11.5)	-	-	Before and after chemotherapy and 4 months later	EORTC-QLQ-C30
Mohammadi S [38], Iran—2013	Cross-sectional	100	47.8(6.7)	I: 8 II: 41 III: 51.3	All patients completed 3 phases of BC treatment, which included mastectomy, chemotherapy, radiation therapy.	Duration of survivorship: 2 years: 25 3 years: 44 4 years: 25 5 years: 6	EORTC-QLQ-C30
Moradi R [39], Iran—2017	Cross-sectional	87	48.25 (11.9)	-	Chemotherapy: 100	After chemotherapy	WHOQOL-BREF
Musarezaei A [40], Iran—2015	Cross-sectional	105	45.3 (4.6)	II: 50	Mastectomy: 100	Underwent mastectomy (at least 1 year and a maximum of 5 years previously)	NMCBRI-Q
Najafi F [41], Iran—2016	Cross-sectional	148	47.6 (10.1)	In situ: 7.1 Local: 45.7 Loco/regional: 40 Advanced: 7.1	Patients completed at least 2 chemotherapy sessions (only those with very small tumors did not undergo chemotherapy).	Time since diagnosis: 17.2 (1.4) months	EORTC-QLQ-C30
Nikamenesh Z [42], Iran—2017	Cross-sectional	42	30-50 years: 69%	-	-	Diagnosis for at least 6 months and under treatment	EORTC-QLQ-C30
Pehlivan S [43], Turkey—2016	Cross-sectional	61	50.37 (11.8)	I: 9.8 II: 59 III: 23 IV: 8.2	Chemotherapy: 19.7 Chemotherapy + Radiotherapy: 80.3	During Radiotherapy	EORTC QLQ-BR23
Rohani C [44], Iran—2015	Case-control	Cases (BC patients): 162 Controls: 210	Cases: 46.1 (9.8) Control: 46.6 (8.4)	0: 2.5 I: 22.8 II:49.4 III: 24.1 IV: 1.2	Chemotherapy: 79 Radiotherapy: 75.3 Hormone therapy: 68.5	Baseline pre-diagnosis phase of BC (T1) and 6 months post pre-diagnosis (T2).	EORTC-QLQ-C30
Safa A [45], Iran—2014	Cross-sectional	92	42.9 (8.7)	-	-	At least 3 months since treatment	QOL-BC
Shakeri J [46], Iran—2016	Cross-sectional	98	47.6 (14)	-	-	Unspecified	LQI
Saatci E [47], Turkey—2007	Cross-sectional	100	48.64 (10.6)	I: 49 II: 51	Chemotherapy: 59 Radiotherapy: 10 Chemotherapy + Radiotherapy: 31	22.6 (24.3) months	FACT-G
Safaee A [48], Iran—2008	Cross-sectional	119	48.27 (11.4)	Well differentiated: 33.6 Moderately differentiated: 42 Poorly differentiated: 24.4	Chemotherapy: 100	Time since diagnosis: < 4 months: 39.5 4-12 months: 34.4 >12 months: 26.1	EORTC-QLQ-C30
Shandiz,F H [49], Iran—2008	Cross-sectional	94	45.20(8.6)	-	Chemotherapy: 100 Radiotherapy: 69.1 Surgery: 100 Hormone therapy: 52.1	During chemotherapy	EORTC-QLQ-C30
Sinaei F [50], Iran—2017	Case-control	Mastectomy group: 45 Reconstruction group: 61	Mastectomy: 50.2 (8.5) Reconstruction: 46.7 (8.1)	-	Surgery: 100 Chemo radiation: Mastectomy: 86.7 Reconstruction: 90	unspecified	EORTC-QLQ-C30 plus QLQ-BR23
Uzun Ö [51], Turkey—2004	Cross-sectional	72	50.1 (9.3)	I: 24 II: 58.3 III: 16.7	Chemotherapy: 100 Radiotherapy: 52.8 Surgery: 100	unspecified	QoLS
Zamanian H [52], Iran—2015	Cross-sectional	224	47.1 (9.0)	-	Chemotherapy: 61.6 Radiotherapy: 40 Surgery: 26.8	Time since diagnosis: 43.8 (37.6) months	FACT-B
Zargani A [53], Iran—2018	Cross-sectional	84	54.7 (10.4)	-	All patients undergoing cancer treatment (surgery, radiotherapy or chemotherapy).	Diagnosis at least 1 year prior	SF-36

Age of breast cancer patients and time since diagnosis of BC are presented as mean (standard deviation), stage of BC I, II, III, and IV, and interventions (chemotherapy, radiotherapy, surgery, and hormonotherapy) are presented as number (percentage). Abbreviations: BR23: Breast cancer specific module. EORTC-QLQ-C30: European Organization for Research and treatment of Cancer—Quality of Life Questionnaire. FACT-B: The Functional Assessment of Cancer Therapy—Breast Cancer. FACT-G: The Functional Assessment of Cancer Therapy—General Questionnaire. LQI: Life quality inventory. NMCBRI-Q: The national medical center and Beckman research institute questionnaire. QoLS: The Quality of Life Scale. SF-36: Short-Form Health Survey Questionnaire. WHOQOL-BREF: WHO questionnaire of quality of life. FAC: fluorouracil, doxorubicin, and cyclophosphamide. TAC: docetaxel, doxorubicin, and cyclophosphamide.

**Table 4:** Newcastle–Ottawa scale for the assessment of the quality of studies included. (a). Quality assessment of cross-sectional studies and longitudinal studies according to the Newcastle–Ottawa scale. (b). Newcastle–Ottawa scale for the assessment of the quality of case-control studies (each asterisk indicates if the individual criterion within the subsection was fulfilled).

Author	Study Design	Selection of Participants	Comparability Based on Design or Analysis	Assessment of Outcome/Exposure	Total
(a)					
Ahmed et al. [21]	Cross-sectional	**	**	*	5(/6)
Ahrafizadeh et al. [22]	Cross-sectional	**		*	3(/6)
Akel et al. [23]	Cross-sectional	**		*	3(/6)
Akin et al. [24]	longitudinal	***		**	5(/9)
Alawadi et al. [25]	Cross-sectional	**	**	*	5(/6)
Albabtain et al. [26]	Cross-sectional	**		*	3(/6)
Almutairi et al. [27]	Cross-sectional	**	**	*	5(/6)
Al Naggar et al. [28]	Cross-sectional	**	**	*	5(/6)
Al Natour et al. [29]	Cross-sectional	**		*	3(/6)
Bayram et al. [31]	Cross-sectional	**		*	3(/6)
Filazoglu et al. [32]	Cross-sectional	**	**	*	5(/6)
Hujeir et al. [33]	Cross-sectional	**		*	3(/6)
Jafari et al. [34]	Cross-sectional	**	*	*	4(/6)
Jassim et al. [35]	Cross-sectional	**	**	*	5(/6)
Khalili et al. [36]	Cross-sectional	**		*	3(/6)
Kiadaliri et al. [37]	longitudinal	***		**	5(/9)
Mohammadi et al. [38]	Cross-sectional	**		*	3(/6)
Moradi et al. [39]	Cross-sectional	***		*	4(/6)
Musarezaei et al. [40]	Cross-sectional	**		*	3(/6)
Najafi et al. [41]	Cross-sectional	***	**	*	6(/6)
Nikmanesh et al. [42]	Cross-sectional	**		*	3(/6)
Pehlivan et al. [43]	Cross-sectional	**		*	3(/6)
Saatci et al. [47]	Cross-sectional	**		*	3(/6)
Safa et al. [45]	Cross-sectional	***		*	4(/6)
Safaee et al. [48]	Cross-sectional	**	**	*	5(/6)
Shakeri et al. [46]	Cross-sectional	**		*	3(/6)
Shandiz et al. [49]	Cross-sectional	**	**	*	5(/6)
Uzun et al. [51]	Cross-sectional	**		*	3(/6)
Zamanian et al. [52]	Cross-sectional	**	**	*	5(/6)
Zargani et al. [53]	Cross-sectional	**		*	3(/6)
(b)					
Bagheri et al. [30]	Case-control	*	*	**	4(/9)
Rohani et al. [44]	Case-control	***	**	**	7(/9)
Sinaei et al. [50]	Case-control	***	**	**	7(/9)

“*” star system: for cohort study: A study can be awarded a maximum of one star for each numbered item within the Selection and Outcome categories. A maximum of two stars can be given for Comparability. For cross-sectional study: A study can be awarded a maximum of one star for each numbered item within the Selection and Exposure categories. A maximum of two stars can be given for Comparability.

**Table 5:** Global health-related quality of life (HRQoL) and its associated factors in women with breast cancer in the Middle East.

Author Name	Global HRQoL Mean (SD)	Determinants of HRQoL	Signs of Association
Ahmed A et al. [21]	50.7 (19.2)	Regular exercise	+
		Multiple tumors	−
		Metastasis	−
		Fever	−
		Age	−
Ahrafizadeh H [22]	87.9 (2.15)	Higher level of education	+
		Higher income	+
		Type of surgery (breast conserving surgery versus mastectomy)	+
		Duration of the disease/diagnosis	+
Akel R [23]	108.7 (18.7)	Iraqi	−
		Stage IV	−
		Monthly income below 1000 USD	−
		Chemotherapy	−
		Anxiety and depression	−
Akin S [24]	Before chemotherapy: 69.2 (21.6) chemotherapy: 53.3 (20.5)	Age (40–50 years)	−
		Married	−
		Employed women	−
		Low income	−
		Obesity	−
		Stage I, II, III,	−
		FAC/FEC and Docetaxel/Paclitaxel AC/EC	−
		Diagnosis since 2–6 months	−
		Breast-sparing surgical procedure	−
Alawadi S [25]	45.3 (15.3)	Social functioning	−
		Sexual enjoyment	−
		Pain	−
		Financial difficulty	+
Albabtain H [26]	CAM = 73.1 (20.2) No CAM = 64.8 (32.7)	Complementary alternative medicine	+
Almutairi K [27]	31.2 (20.5)	Pathological staging	−
Al-Naggar R [28]	< 55 years = 83.2 (22.8) ≥ 55 years = 77 (21.6)	Lower income	−
		>2 years after diagnosis	−
		Under radiotherapy	+
Al-Natour A [29]	79.9 (18. 1)	Spiritual wellbeing	+
Bagheri M [30]	73.3 (11.2)	Body image, Appearance evaluative, Appearance orientation, Fitness evaluative, Fitness orientation, Subjective weight, Body satisfaction	+
Bayram Z [31]	63.8 (16.4)	Literate, Homemakers, ECOG2, Stage IV, Chemotherapy agents (gemcitabine+cisplatin).	−
Filazoglu G [32]	-	Physical component score:	PCS:
		Stage of Cancer	−
		Time since operation	+
		Time since diagnosis	+
		Social support	+
		Problem solving coping	+
		Mental component score:	MCS:
		Type of treatment	−
		Stage of Cancer	−
		Time since operation	+
		Social support	+
		Problem solving coping	+
		Helplessness coping	−
Hujeir H [33]	59.6 (29.0)	Psychological symptoms	−
		Physical symptoms	−
		Total memorial symptom assessment scale	−
Jafari N [34]	41.4 (18.0)	Social functioning	+
		Pain	−
		Arm symptoms	−
		Spiritual well-being	+
Jassim G [35]	63.9 (21.3)	Advanced stage	−
		Menopause	−
Khalili N [36]	60.3 (21.10)	Emotion focused coping strategy	+
		Affective interference of pain	−
Kiadaliri A [37]	Baseline: TAC: 69.3 (0.9) FAC: 69.4 (1.5)	Having children	+
		Being married	−
		TAC treatment	−
Mohammadi S [38]	68.1 (17.7)	Nutritional status (well-nourished)	+
Moradi R [39]	75.9 (15.2)	Economic status	+
		Self-efficacy	+
Musarezaei A [40]	Weak HRQoL 16.36% Moderate HRQoL 58.48% Good HRQoL 25.15%	Higher level of education	+
		Fatigue	−
Najafi F [41]	43.86 (23.5)	Older Age	+
Nikamenesh Z [42]	10.78 (2.4)	Self-regulation	+
Pehlivan S [43]	55.1 (10.8)	Age, RTCS, Functional, Symptom	+
Rohani C [44]	Baseline:	Sense of coherence	+
	BC group: 58.1 (20.1)		
	Control group: 70.1(21.6)		
	6 months post pre-diagnosis:	Education	+
	BC group: 68.7 (18.5)		
	Control group: 72.4 (18)		
Safa A [45]	Average QoL: 87% High QoL: 13%	Age	+
		Disease duration	+
		Fatigue	+
		Interval between diagnosis and treatment	−
Shakeri J [46]	70.02 (14.4)	Depression	−
Saatci E [47]	76.1 (14.8)	Unmet needs	+
		Working women	+
Safaee A [48]	64.9 (11.4)	Grade of tumor	−
		Occupation	+
		Post menopause	+
		Financial difficulties	−
		Dyspnea	−
Shandiz,F H [49]	71.4 (22.3)	Older age	−
		High social status	+
		No radiotherapy	+
		Receiving hormone therapy	+
		Fatigue	−
		Dyspnea	−
Sinaei F [50]	Mastectomy group: 57.1 Reconstruction group: 72.5	Breast reconstruction surgery more positive effect on QoL than Mastectomy	+
Uzun Ö [51]	147.6 (24.0)	Education	+
		Employed women	+
Zamanian H [52]	105.0 (22.7)	Positive religious coping	+
Zargani A [53]	73.1 (9.2)	Religiosity	+

Abbreviations: AC: Doxorubicin/Cyclophosphamide. CAM: Complementary alternative medicine. EC: Epirubicin/Cyclophosphamide. ECOG: Eastern cooperative oncology group performance score. FAC = 5-fluorouracil, doxorubicin, and cyclophosphamide. FEC: Fluorouracil, Epirubicin, Cyclophosphamide. RTCS: Radiation therapy comfort scale. TAC: Docetaxel with doxorubicin and cyclophosphamide. +: signifies a positive association between determinants and HRQoL, determinant develop a better HRQoL.: signifies a negative association between determinants and HRQoL, determinant develop a lower HRQoL.
